# Enhancing reproducibility in developmental EEG research: BIDS, cluster-based permutation tests, and effect sizes

**DOI:** 10.1016/j.dcn.2021.101036

**Published:** 2021-11-12

**Authors:** Marlene Meyer, Didi Lamers, Ezgi Kayhan, Sabine Hunnius, Robert Oostenveld

**Affiliations:** a Donders Institute for Brain, Cognition and Behavior, Radboud University, Nijmegen, NL, USA; b NatMEG, Karolinska Institutet, Stockholm, SE, USA; c Department of Psychology, University of Chicago, Chicago, IL, USA; d Radboud University Library, Radboud University, Nijmegen, NL, USA; e Department of Developmental Psychology, University of Potsdam, Germany; f Max Planck Institute for Human Cognitive and Brain Sciences, Leipzig, Germany

**Keywords:** EEG, reproducibility, cluster-based permutation test, effect size, BIDS

## Abstract

Developmental research using electroencephalography (EEG) offers valuable insights in brain processes early in life, but at the same time, applying this sensitive technique to young children who are often non-compliant and have short attention spans comes with practical limitations. It is thus of particular importance to optimally use the limited resources to advance our understanding of development through reproducible and replicable research practices. Here, we describe methodological approaches that help maximize the reproducibility of developmental EEG research. We discuss how to transform EEG data into the standardized Brain Imaging Data Structure (BIDS) which organizes data according to the FAIR data sharing principles. We provide a tutorial on how to use cluster-based permutation testing to analyze developmental EEG data. This versatile test statistic solves the multiple comparison problem omnipresent in EEG analysis and thereby substantially decreases the risk of reporting false discoveries. Finally, we describe how to quantify effect sizes, in particular of cluster-based permutation results. Reporting effect sizes conveys a finding’s impact and robustness which in turn informs future research. To demonstrate these methodological approaches to data organization, analysis and report, we use a publicly accessible infant EEG dataset and provide a complete copy of the analysis code.

## Highlights

• Methods for enhancing reproducibility in developmental EEG research.
• Tutorial for converting EEG data into BIDS to adopt FAIR data sharing principles.
• How to use cluster-based permutation testing to analyze developmental EEG data.
• How to quantify effect sizes, particularly of cluster-based permutation results.

## 1 Introduction

Electroencephalography (EEG) is a particularly useful and effective technique to assess brain activity in developmental populations. Other neuroimaging techniques, like fMRI, PET, TMS or MEG, typically require participants to keep still over a prolonged period of time, involve unpleasant procedures or are expensive. In contrast, EEG is a non-invasive, infant-friendly method to measure neural activity with high temporal resolution. As evident from the growing body of developmental research using EEG, valuable insights can be gained on the emergence of processes in a multitude of domains including cognitive, social, and clinical domains (e.g., Bell and Cuevas, 2012; Bosl et al., 2011; Braithwaite et al., 2020; de Haan, 2013; Endedijk et al., 2017; Friederici, 2005; Marshall et al., 2004). At the same time, developmental research with EEG comes with substantial challenges, be it for research investigating event-related potentials (ERP) or frequency information (Bell and Cuevas, 2012, Hoehl and Wahl, 2012, van der Velde and Junge, 2020). For instance, the quality of developmental EEG data is limited because of short preparation and recording times due to short attention spans especially of infants. Less tolerance for wearing the EEG cap, excessive movement during recording, and higher drop-out rates in infants and young children compared to adult participants can also lead to poorer data quality. As such, developmental EEG data are both valuable and costly. To optimally use these limited resources and increase their impact for future research, properly applied and reported statistical methods are crucial (Klapwijk et al., 2020). Moreover, sharing data and analysis code with the scientific community further contributes to reproducible science. Here, we describe three methods for organizing, analyzing and reporting developmental EEG data to support reproducibility and replicability^1^ of developmental EEG research. In particular, we show 1) how the Brain Imaging Data Structure (BIDS; Gorgolewski et al., 2016) can be used to organize developmental EEG data, 2) how cluster-based permutation testing can be implemented to analyze developmental EEG data (Maris and Oostenveld, 2007), and 3) how effect sizes for cluster-based permutation results can be calculated and reported in a meaningful way (Flournoy et al., 2020).

### 1.1 Organizing developmental EEG data in BIDS

Sharing developmental EEG data and the corresponding analysis code according to the FAIR data sharing principles (Wilkinson et al., 2016) allows for better interoperability, reproducibility, and allows others to better evaluate the respective research (Peng, 2011). In addition, making developmental EEG data available for reuse ensures efficient usage of the limited resources and stimulates novel data- and hypothesis-driven research. The potential of reusing data is hampered when data is shared in idiosyncratically organized formats, but for a long time, clear standards for data sharing have been missing. BIDS (Gorgolewski et al., 2016; https://bids.neuroimaging.io) is a standardized format, widely used within the field of neuroscience and across multiple techniques, like MRI, PET, EEG, MEG and NIRS (e.g., Knudsen et al., 2020; Niso et al., 2018, Pernet et al., 2019), yet still only rarely used amongst developmental scientists. Organizing developmental EEG data in BIDS helps making data accessible for collaborating researchers within the same lab environment. When that data is furthermore publicly shared it also enhances reuse of the data by researchers from other labs. It even solves the well-known problem of making sense of one’s own collected data after several years have passed after data collection. Here, we describe how to transform data from an infant EEG study into BIDS and which data to include in BIDS. For instance, BIDS contains raw data as well as metadata. Metadata, such as information on which EEG marker refers to which stimulus, makes the raw data interpretable. As described in detail in our Methods section, BIDS entails a clear folder hierarchy and specific naming conventions. Note that BIDS thus offers a way of organizing developmental EEG data which facilitates but does not dictate the sharing of data.

### 1.2 Analyzing, reporting and interpreting developmental EEG data

EEG data consist of a high-dimensional representation of brain activity. Therefore, statistical testing for effects between experimental conditions at multiple channels, latencies, and frequencies often results in a multiple comparisons problem. When the large number of statistical comparisons is not explicitly dealt with, this dramatically increases the probability of false positives. This probability is also called family-wise error rate (FWER). Previous developmental EEG studies have often addressed this issue by reducing the dimensions of the data based on a priori assumptions. For instance, the mean amplitude of a signal from pre-selected channels, time points and frequencies has been statistically compared using univariate statistics like F- or *t*-tests (e.g. Hoehl and Wahl., 2012). However, reducing the data in such a way is not always possible due to a lack of a priori information, in particular in developmental populations in which the topography, latency, and spectral distribution can differ strongly from adult populations and across differen age groups (see e.g., Meyer et al., 2016). Moreover, such a procedure artificially reduces the richness of high-dimensional EEG data. One solution for the multiple comparison problem, while preserving the high dimensionality of the EEG data, is to use cluster-based permutation tests (Maris and Oostenveld, 2007). Initially, such an approach was used in MRI research with adults where it is known as cluster mass test (Bullmore et al., 1999). This by now well-established method for fMRI, MEG, and EEG analysis of adult data (e.g., Pernet et al., 2015) is still used seldom for developmental EEG (but see e.g., Meyer et al., 2020; Meyer and Hunnius, 2021; Sommer et al., 2021). The cluster-based permutation test not only corrects for the multiple comparison problem and thereby reduces false positive results, it also reduces the potential for false negative effects. Especially for developmental EEG data that has limitations in data quality and number of participants, a sensitive statistical test balancing false positive and false negative results can help maximize power do detect true effects and in turn enhance reproducibility.

#### 1.2.1 Cluster-based permutation test

The basic idea of the cluster-based permutation test consists of using a permutation or randomization test to approximate the probability distribution of a nonparametric statistic (Maris and Oostenveld, 2007). It involves 1) reassigning the trials of different conditions in a random manner, 2) calculating a test statistic, 3) repeating these computations to create a permutation distribution, and 4) determining where in this distribution the statistic falls for the observed data under the original conditions. Since the number of possible permutations is often larger than computationally tractable, it is common to use a Monte Carlo estimate of the permutation distribution by randomly sampling a subset of all possible permutations. Importantly, in the cluster-based permutation test comparable effects over temporally, spatially and spectrally adjacent samples are clustered (Maris and Oostenveld, 2007). A clear advantage of using nonparametric tests, like the cluster-based permutation test, is that any test statistic can be used to investigate the difference between different conditions or the relationship between data and an independent variable. Thereby the statistical sensitivity is increased while also mitigating the risks of multiple comparisons and minimizing false negatives (Maris and Oostenveld, 2007). Moreover, parametric tests often require normally distributed error terms. In contrast to this, nonparametric tests do not rely on this assumption and are therefore more universally applicable.

#### 1.2.2 Effect sizes

Effect sizes are a quantification of how large an observed effect is. As such, effect sizes speak to the robustness of a finding and are important input, for instance to inform future research through power analysis. While the importance of reporting effect sizes is increasingly acknowledged in the field of developmental cognitive neuroscience (Flournoy et al., 2020, Klapwijk et al., 2020), both for hypothesis-driven and exploratory data analysis, it is less obvious how to actually quantify and report an effect size, particularly for results of nonparametric tests like the cluster-based permutation test (Computing and reporting the effect size, 2021). One of the effect sizes often used and reported is Cohen’s d, which quantifies the standardized difference between means. Yet, the cluster-based permutation test is *nonparametric* and computes the probability of exchangeability under the null hypothesis that the data in the two conditions comes from the same distribution; it does not compute the probability of a specific parameter, such as the mean over subjects, being the same. Consequently, if following the rejection of the null hypothesis we want to quantify the effect size using the mean, it is not directly obvious which data to include in the computation of that mean, i.e., whether to compute the average effect size over the cluster, or the effect size over the average of the cluster, or whether to report the maximum effect size within a cluster for a specific channel, time, and frequency combination. Here, we demonstrate and discuss the different approaches to compute and report effect sizes.

### 1.3 The current paper

**Fig. 1:**
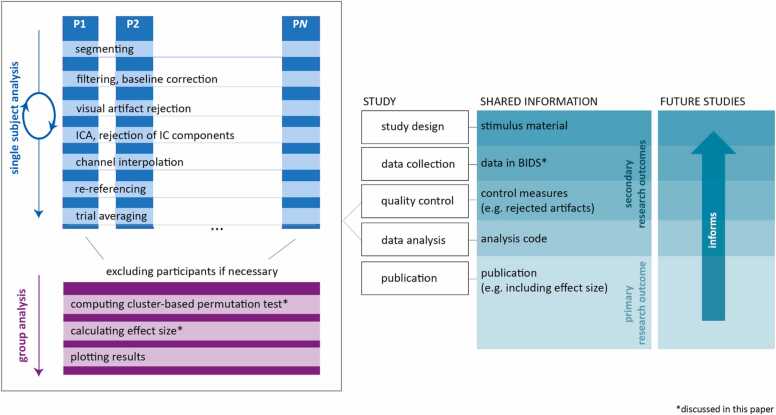
Schematic overview showing the analysis workflow (left) that first loops over N participants in single subject pre-processing steps and concludes with a group analysis; the elements of a specific developmental EEG study (middle); and the information that can be shared from a developmental EEG study to inform future research and foster reproducibility (right). Asterisks indicate the topics discussed in this paper.

The purpose of this paper is to offer concrete examples on how to organize and analyze developmental EEG data, and how to report results from developmental EEG data, with focus on maximizing reproducibility and value for future research (see Fig. 1, for an overview). More specifically, we provide a tutorial on how to 1) transfer an example raw infant EEG dataset into BIDS, 2) use cluster-based permutation testing to compare infant EEG data across different conditions, and 3) calculate and report effect sizes, particularly for cluster-based permutation tests. For this purpose, we make use of an event-related EEG dataset that was collected from 9-month-old infants (Kayhan et al., 2019). The analyses focus on ERPs and we assume for the analyses presented here that there were no a priori hypotheses on where and when to expect an effect. While we focus on ERPs here, the approach outlined in this paper is equally applicable to developmental research examining the EEG in the spectral domain, or conducting time-frequency analysis. Here, we consider clusters based on channels and time, but when studying power spectra one could also form clusters over channels and frequencies, or clusters over channels, time, and frequencies with time-frequency analysis (e.g., wavelets). Both a static copy of the analysis code and the example infant EEG data are made available at https://doi.org/10.34973/gvr3–6g88 and https://doi.org/10.34973/g4we-5v66. Furthermore, the analysis code is maintained on GitHub (https://github.com/Donders-Institute/infant-cluster-effectsize).

## 2 Methods

### 2.1 Example infant EEG dataset

The infant EEG dataset used here was collected to investigate infants’ evoked responses to different types of violations in a predictable sequence of stimuli (Kayhan et al., 2019). For the current purpose, we focus on a simple comparison of repeated stimuli in a sequence versus stimuli violating that audio-visual regularity. Stimuli which draw infants’ attention, for instance by violating their expectation, elicit a negative ERP potential over fronto-central channels, called negative central (Nc; e.g., Jeste et al., 2015; Webb et al., 2005). Since we did not have a sufficiently specific a priori hypothesis on precisely where and when to expect a difference between experimental conditions (for variations in latency and amplitude see e.g., Köster et al., 2021; Richards et al., 2010; Stets and Reids, 2011; van Hoogmoed et al., 2013; Webb et al., 2005), we tested all channels and latencies and deal with the multiple comparison problem by making use of the cluster-based permutation test. We demonstrate in the following how to organize infant EEG data in BIDS, allowing a variety of research questions to be addressed from this standard data representation, and we take this classic comparison to exemplify how to perform a cluster-based permutation test for ERPs, and how to quantify and report effect sizes for the results. The anonymized infant EEG dataset used as example for this tutorial is available from the Donders Repository together with a static copy of the analysis code (https://doi.org/10.34973/gvr3–6g88 and https://doi.org/10.34973/g4we-5v66). Moreover, the analysis code is maintained on GitHub (https://github.com/Donders-Institute/infant-cluster-effectsize).

#### 2.1.1 Participants

The dataset comprises EEG recordings of 59 infants who were at 9-months of age (M = 272.78 days, range: 251–289 days, 31 girls). Due to insufficient artifact-free trials 11 participants had to be excluded such that 48 participants were included in the final analysis (see *Analyzing developmental EEG data* in the Methods section for more details). Participants were recruited from a database of families living in and around a middle-sized city in the Netherlands. All infants were born full-term and had no indications of atypical development. For their participation, families received a children’s book or 20 euros. The study was approved by the regional ethics board (CMO 2012/012-NL39352.091.12).

#### 2.1.2 Stimuli and study design

In the study, the infants’ neural response to audio-visual stimuli was measured. Stimuli consisted of simple sounds and cartoon images of bees of different colors, and two shape images: a triangle and a circle. All image and audio files are available in the data repository. The bee images were presented at one of eight locations positioned on a circle around the center of the screen. The shape stimuli and fixation cross images were presented at the center of the screen. For the purpose of the current tutorial, we focus on the contrast between the repeated bee stimuli (in the following referred to as *standard* stimuli) and the two shape stimuli that interrupted the sequence of bees (in the following referred to as *oddball* stimuli). For more details on the procedure see Kayhan et al. (2019). In total, standard stimuli were presented 90 times and oddball stimuli 50 times. Each image was accompanied by a unique sound, and image-sound associations were counterbalanced across participants.

The stimuli were presented by means of Presentation Software (Neurobehavioral Systems, Inc., Albany, CA) as follows. After a 500 ms grey screen, a fixation cross was presented for 1000 ms. At the start of a sequence, a standard stimulus was presented for 1500 ms. The same standard stimulus (with matching position and color) was then presented multiple times, interleaved with a grey screen and the fixation cross image until the sequence was interrupted with an oddball stimulus (presented for 1500 ms). After presentation of the oddball stimulus the same sequence of standard stimuli was either continued (50% of the time) or a new sequence was initiated with a standard stimulus of a different color, position and sound. Together, stimulus presentation took maximally 9 min.

#### 2.1.3 EEG recordings

Infants’ EEG was recorded from 32 active electrodes arranged in the standard 10–20 layout using an infant-sized actiCap (actiCap, Brain Products GmbH, Gilching, Germany). The signal was digitized at 500 Hz, using a BrainAmp DC EEG amplifier and a band-pass filter (low cut-off at 0.1 Hz and high cut-off at 200 Hz). The left mastoid was used as online reference, with AFz as ground. During the recording, infants sat on their parent’s lap in an electrically shielded testing room. Parents were instructed to keep any interaction with their child to a minimum. Additionally, the sessions were video-recorded. Note that identifiable data such as video recordings are not part of the accessible data collection for privacy reasons.

### 2.2 Organizing developmental EEG data in BIDS

We used the Brain Imaging Data Structure (BIDS, Pernet et al., 2019) to organize the raw infant EEG data for maximal interoperability and reuse according to FAIR principles (Wilkinson et al., 2016). The conversion of the original “source data” into the BIDS format was done using MATLAB (version R2019a, Mathworks, Inc.) and the open-source FieldTrip toolbox (Oostenveld et al., 2011). In the following, we highlight several aspects of BIDS relevant to developmental EEG. The full specification of the BIDS standard is available online (https://bids.neuroimaging.io).

#### 2.2.1 Data and metadata

Depending on the specifics of the study and the data that were recorded in addition to EEG (e.g., eye-tracking, video, questionnaires etc.), several files must be included in BIDS, while others are optional additions to the data collection. For a detailed description, see https://bids-specification.readthedocs.io. Raw EEG data must be stored in an open file format. Metadata that accompany the raw data provide the required information to interpret and reuse the EEG data. For example, this includes information about the EEG acquisition system and settings, the time and interpretation of markers that describe stimuli and events that were recorded synchronously with the EEG, and the age and gender of each participant. Metadata that are represented in a tabular form are stored using Tab Separated Values (.tsv) files and metadata that are structured otherwise are stored in JavaScript Object Notation (.json) files. For all tabular information it is possible to include a data dictionary that further explains the data. For instance, the column “age” in the participant table (participants.tsv) is complemented in the data dictionary with the information that its unit of measurement is in “days”. It is advised to test whether the required data and metadata are present and formatted properly using the BIDS validator (https://github.com/bids-standard/bids-validator/), which allows checking the BIDS representation in a web browser without uploading any data.

BIDS distinguishes between source data, raw data and derivatives. The source data are the non-standardized data prior to converting: the EEG data, the video data, but also the logfiles from the Presentation Software, lab notes, or an Excel spreadsheet with information about the participants. It is possible to share (part of) the source data, but not required. The raw data according to BIDS is the data in a standardized format without any processing. To maximise the potential for data reuse, the task description and stimulus events are given such that analyses that are different from the original research questions can also be addressed. Derivatives are the results of processing the raw data, such as the filtered, segmented, and cleaned data, but also simple tabular files containing information about the channels and trials that were excluded from further EEG processing. Derivative data represent the efforts and the choices made by the researcher who has analyzed the data and can be shared with a BIDS data collection. The derivative data needs to be kept separate from the non-processed raw data. Since for the analysis of the original study specific and possibly subjective choices may have been made (e.g., selection of trials) derivatives might not be appropriate for a secondary analysis. For instance, for a researcher wanting to reuse shared data to analyze the neural signature of blinks in developmental EEG data it would be impossible to extract that information if only processed and artifact-corrected EEG data were shared.

#### 2.2.2 Folder hierarchy

**Fig. 2:**
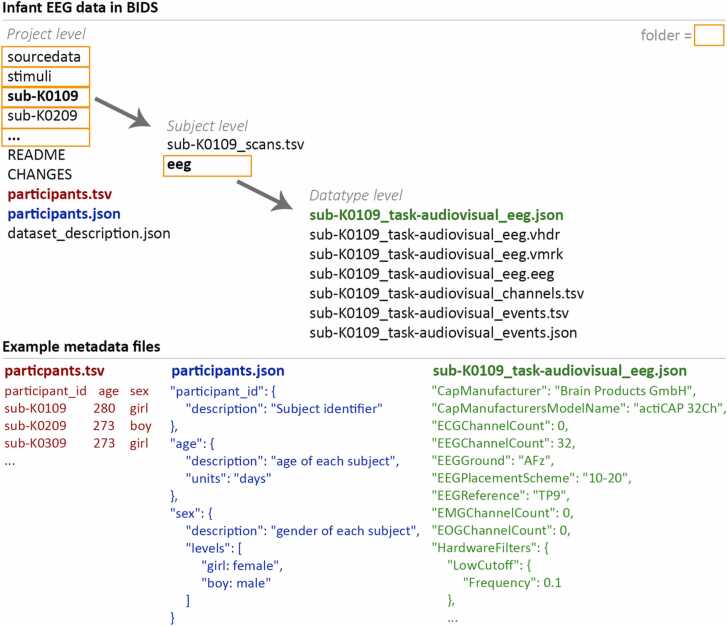
Example BIDS folder structure and metadata information for the example infant EEG dataset.

Generally, data in BIDS are arranged in a folder hierarchy (project / subject / session / datatype) starting with a project level that contains information about the entire project. In this case, for instance, an overview of the participants is included, as well as a folder with the stimulus material. Below the project level is the subject level, which contains information specific to each participant such as any measurements pertaining to this specific participant. This is optionally followed by the session level separating data from multiple sessions attended by the participant (if applicable). This is particularly important in longitudinal research. Since in the current infant EEG dataset all participants underwent only a single testing session, we omitted this level. The last level specifies the datatype. In our case, this only entails EEG, but other measures such as behavioral measures or eye-tracking data can be added, as well as other recordings of brain data, such as MEG, anatomical or functional MRI, and so forth. Fig. 2 illustrates the BIDS folder hierarchy used in the current infant EEG example dataset.

#### 2.2.3 Naming conventions

BIDS adheres to specific file naming conventions. For instance, the data files of each participant always start with ‘sub-’ followed by a unique identifier, for example ‘sub-02′. If there are multiple sessions, the file of each participant and session must also contain ‘ses-’ followed by the session number. In event-related EEG recordings, one has to additionally specify ‘task-’ followed by a short description of the task. In our event-related infant EEG study, the file of one infant’s EEG data was thus called ‘sub-K0109_task-audiovisual_eeg’.

#### 2.2.4 Example infant EEG BIDS dataset

In our example Script Section 1 (corresponding to file ‘do_convert_data_to_BIDS.m’), we demonstrate how to convert the original source data from an infant EEG dataset to the raw representation according to BIDS. Note that the original source data on which the conversion to BIDS format is based contains identifiable information and therefore cannot be shared publicly. For this reason, this script cannot be executed on the original, identifiable data by the reader. Still, it exemplifies the important steps for data curation and converting developmental EEG data to BIDS. The result of the BIDS transformation is shared in the Donders Repository (https://doi.org/10.34973/gvr3–6g88) and contains all data and metadata required for the analysis. By navigating through this dataset, the folder structure, naming conventions and file types used for a typical event-related developmental EEG study can be explored. Based on this dataset, all scripts containing pre-processing and analysis (i.e. Script 1.1, 2) can be run and tested by the reader.

### 2.3 Analyzing developmental EEG data

#### 2.3.1 Preprocessing single subject EEG data

The analysis code regarding the preprocessing of single subject EEG data is reflected in Script Section 1.1, corresponding to file ‘do_singlesubject_analysis.m’. The EEG data were segmented into 1500 ms trial epochs, time-locked to the onset of the standard and oddball stimuli, with a 500 ms pre- and 1000 ms post-stimulus period (Script Section 1.2). To include only the repeated standard stimuli, the first standard stimulus of a sequence and the stimulus immediately following the oddball stimulus were excluded from the analysis. Consistent with Kayhan et al. (2019), the trial epochs were band-pass filtered between 1 and 30 Hz (with 5-sec padding) and baseline correction was applied on the entire epoch window (Script Section 1.3). Subsequently, artifact rejection was conducted in three steps, blind to condition (Script Section 2.3). In a first step, all trials and channels were inspected visually to manually exclude those trials and channels that contained large artifacts. In a second step, independent component analysis (ICA) was performed to detect and correct for artifacts, for instance caused by eye-movements or heartbeat. The independent components were then visually inspected and those containing artifacts rejected from the data. In a third step, another pass of visual artifact rejection was performed to discard any remaining trials or channels with artifacts. Rejected bad channels were then interpolated. After artifact rejection, the cleaned EEG data were re-referenced to linked mastoids and the event-related trials were averaged per condition for each participant (Script Section 2.4 and 2.5). It should be noted that it is beyond the scope of this paper to provide a standard for pre-processing pipelines of developmental EEG data. Examples of pre-processing pipelines for developmental EEG data are available elsewhere (e.g., Debnath et al., 2020; Gabard-Durnam et al., 2018).

#### 2.3.2 Analyzing group data

The analysis code on analyzing group data is reflected in Script Section 2, corresponding to file ‘do_group_analysis.m′. Before running statistical analyses on the pre-processed data, any participant for whom more than 70% of the trials were rejected was excluded from the analysis (Script Section 2.1). This was based on the rationale that with the removal of 70% of the trials (i.e. a reduction to 0.3 times N) the expected standard error of the mean (SEM) in this within-subject design is approximately 2 times larger than the SEM over the full N. As a result, 11 participants were excluded from further analysis, leaving 48 participants for the group analysis. The remaining participants had on average 45 standard (range 11–78 trials) and 24 oddball trials (range 2–43 trials) left in the analysis. We then visualized the ERP data across participants for the different conditions by averaging data to a grand average ERP separately per condition (standard, oddball; Script Section 2.2). To statistically test condition differences while preserving temporal and spatial information of the data, we conducted a ***cluster-based permutation test*** (see TEXT Box 1) including all channels and time points (Script Section 2.3). In general, if there is prior information as to when and where to expect an effect this may inform a selection of channels and time points to include in a cluster-based permutation test.

Box 1 **Cluster-based permutation test.**

• *Massive-univariate statistic:* In our example, we compare ERPs over two within-subjects conditions, standard and oddball, and therefore chose a dependent samples *t*-test as statistic of interest. The statistic can be chosen by the researcher, making the cluster-based permutation test versatile and usable for different types of comparisons. For instance, two between-subjects conditions could be compared using an independent samples *t*-test. Alternatively, one might want to test for a (linear) effect, like in the current dataset one might want to investigate whether the number of stimulus repetitions in a sequence had a linear effect on the neural signal. In this case, dependent samples regression coefficients can be used. The FieldTrip toolbox implements several test statics. For an overview, see functions starting with the name ‘ft_statfun’ (https://github.com/fieldtrip/fieldtrip/tree/master/statfun). However, researchers are not limited by this selection but can implement any statistic that may be relevant for their own research.
• *Cluster threshold:* The cluster threshold specifies which data points to select for forming a cluster. Here, we use the dependent samples t-values exceeding the typically critical value. Note that the choice of the threshold determines how many data points are included in a cluster: A low threshold will yield larger clusters, while a high threshold will yield smaller clusters. Since this holds true for both the observed and randomly shuffled data the threshold does not influence the validity of the cluster-based permutation test (Maris and Oostenveld, 2007). The specific threshold might, however, make the test less sensitive in specific cases. For instance, when using a high threshold, effects that are small in amplitude but extend over multiple channels and a longer period might remain undetected. To circumvent this problem of having to specify an optimal but also rather arbitrary threshold, threshold-free cluster enhancement can be used (TFCE, Smith and Nichols, 2009).
• *Quantifying a cluster:* There are multiple ways by which clusters can be quantified, for instance, by the sum of values within the cluster or by combining cluster size and intensity in the weighted cluster mass measure. We chose the sum of t-values in the cluster as the quantification as suggested by Maris and Oostenveld (2007). When the sum of t-values in a cluster is positive, we will refer to it as a positive cluster, and idem for negative clusters.
• *Comparing clusters:* To compare clusters, the largest cluster in the observed data and the largest cluster in each random shuffle are selected. Next, the proportion of times is determined for which the sum of t-values of the largest cluster of our observed data is larger than the sums from the randomly shuffled data. If this proportion falls below a critical level (typically.05), the null-hypothesis of exchangeability of the data over the two conditions is very unlikely and rejected, hence the test result is significant. Consequently, we conclude that the data in the two conditions is different. Note that this holds for the largest negative and largest positive cluster, respectively (see also *Positive and negative clusters*). Focusing on the largest cluster as determined by the maximum sum over the cluster may limit the detection of smaller effects in EEG data. Importantly, however, it ensures that the effects detected by the test are corrected for the FWER and therefore reduces the chance of false discoveries.
• *Positive and negative clusters:* When determining where the derived test statistic of the observed data falls in the permutation distribution, one might want to test one tail or both tails of the distribution. If there is no a priori hypothesis specifying the direction of the effect, two-tailed testing is most appropriate. In that case, both the largest positive cluster and the largest negative cluster are compared to the permutation distributions of the largest positive and negative clusters, respectively. Since this involves two tests, we correct for multiple comparison using Bonferroni correction, either by dividing alpha by two or by multiplying the estimated Monte Carlo p-values by two. Here, we corrected the Monte Carlo p-values.
• *Number of randomizations:* The number of Monte Carlo randomizations determines the accuracy and resolution of the permutation distribution (Maris and Oostenveld, 2007). Therefore, the larger the number of randomizations, the better. In principle, this number is limited only by the number of unique permutations in the experimental design and the number of participants, but in practice it also depends on the computing power, number of channels and timepoints, and time available for the analysis. In a within-subjects design with two conditions, for instance, there are 2 ^n^ unique permutations, with n being the number of participants. For 10 participants that results in 1024 unique permutations, and for 16 participants this number rises to 65536. Since the processing time scales with the number of randomizations, it is possible to estimate the maximal number for which the computations are still feasible. For this, we first execute the test – without looking at the resulting p-value – with a relatively small number of randomizations (e.g., 1000). The time this takes can then be used to estimate a number of permutations that is practically feasible. Note that when making use of such a procedure to estimate the processing time, it is important not to inspect the test results each time, as one might be tempted to stop at a moment when it were significant, which would constitute p-hacking (Nuzzo, 2014). As a rule of thumb, we suggest not to specify less than 1000 randomizations.

Cluster-based permutation testing relies on the underlying structure in the EEG data in which – with sufficiently dense spatial and temporal sampling – neural sources are visible on multiple channels and the activity extends over multiple samples (milliseconds). Several parameters can affect the formation of clusters, and hence, the sensitivity of the cluster-based permutation tests (TEXT Box 2).

Box 2 **Potential factors that influence the formation of clusters.**

• *Temporal and spectral smoothing:* Steps during pre-processing that smoothen the developmental EEG data, like low-pass filters to remove high frequencies from the signal, can affect the test sensitivity. The more smoothened the signal is, the more likely will adjacent data points be detected as part of the same cluster. Thus, pre-processing steps, like filtering and interpolation of bad channels, can improve the sensitivity.
• *Specifying neighbors in channel layout:* The electrode density and layout and the specifications of which channels are regarded as neighbors are essential. A channel with many neighbors is more likely to be included in a cluster, and hence the statistical sensitivity of a cluster-based test is not uniform over the scalp. Channels corresponding to electrodes at the edge of the EEG cap, like TP10, will in general have fewer neighbors than a channel in the center, like Cz, and hence on Cz (and its neighbors) an effect would show more easily than on TP10. To make the sensitivity as spatially homogenous as possible, a uniform and symmetric specification of neighbors is desired. Generally, the neighborhood is bidirectional, in other words, two channels are always regarded as neighbors of each other. Different standard neighborhood templates are provided for common EEG layouts. Also, several automatic strategies are available to define neighbors from scratch. For instance, by defining electrodes with a certain distance as neighbors, or by using triangulation, neighbors can be specified. Since there can be subtle differences between different recording setups, even when based on the same electrode placement scheme (e.g., 10–20), it is advisable to plot the selected neighbors to ensure that the definition of neighbors is accurate, symmetric, and homogenous for the specific electrode layout used in one’s study.

**Fig. 3:**
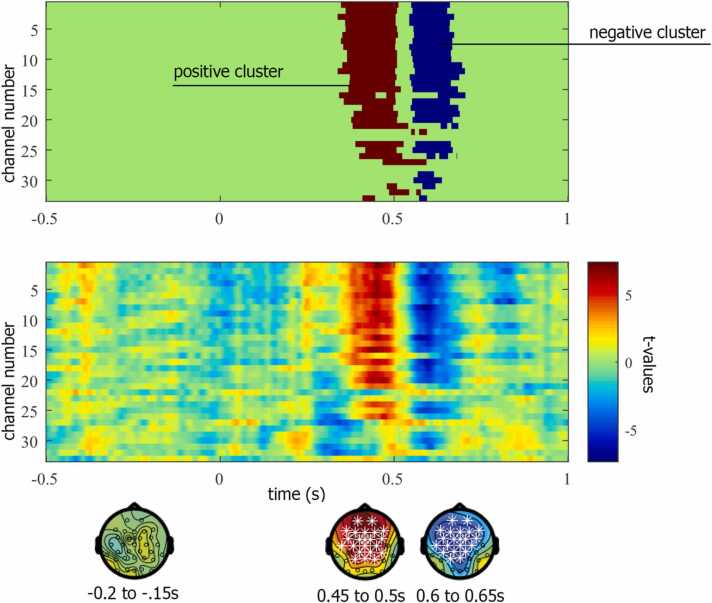
Illustration of the results of the cluster-based permutation test for the example infant EEG data. Top panel: largest positive and negative clusters represented in space (channels) and time (seconds). Middle panel: t-values of the dependent samples *t*-test represented as a function of space (channels) and time (seconds). Topographic distributions are represented for three time windows including a baseline window (−0.2 s to −0.15 s), the positive cluster (around 0.45–0.5 s) and the negative cluster (around 0.6–0.65 s) with white stars reflecting channels that fall into the cluster.

**Fig. 4:**
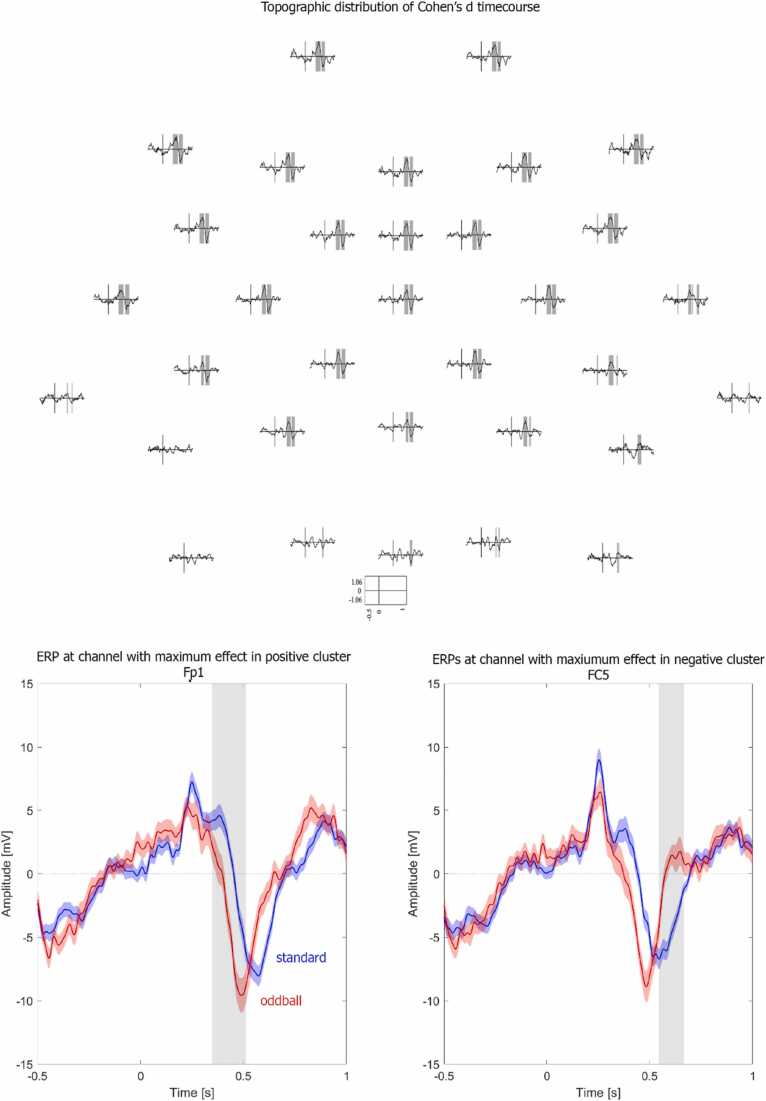
Illustration of effect size. Top: Topographic distribution of Cohen’s d across time. Shaded areas represent data points that fall into the negative or positive cluster. Bottom: Event-related potentials of standard (blue) and oddball (red) conditions at the channel with the maximum effect size of the positive (left) and negative (right) cluster. The grey shaded areas reflect the time window falling into the positive (left) and negative (right) cluster. Shaded areas around the ERPs represent + /- one standard error of the mean.

In our example analysis, the cluster-based permutation test detected two clusters in the data, one for which the quantification of the cluster (i.e. the sum of t-values) was positive (higher amplitude for the standard vs. oddball) and one for which it was negative (lower amplitude for the standard vs. oddball). Fig. 3 illustrates the spatial and temporal distribution of the two clusters (Script Section 2.3.2). The largest positive and negative cluster both have a probability of *p* < .001, leading us to conclude that the null-hypothesis of exchangeability of the data over the two conditions is unlikely. Thus, we accept the alternative hypothesis that the data are different. See also Fig. 4 for a complementary illustration of the ERP findings.

#### 2.3.3 Calculating effect sizes

Next, we estimated the size of the observed effects. Hypothesis-driven research using pre-defined statistical testing on an average over a pre-defined channel, time-window and frequency band may estimate an effect size like Cohen’s d for this pre-defined set of parameters. Here, we did not have a specific a priori hypothesis and therefore made use of the cluster-based permutation test. For the results of the cluster-based permutation test, however, there is no such clear-cut effect size measure, since several different measures could be considered useful and informative (Computing and reporting the effect size, 2021). To illustrate which effect size measures entail what type of information, we calculated several measures of interest (Script Section 2.4). More precisely, we calculated three effect size measures, each based on slightly different parts of the data, and thereby differing in their information content. For all three options, we used Cohen’s d as estimate separately computed for the positive and negative clusters in our infant EEG data.

##### 2.3.3.1 Option 1. Effect size: average over cluster (Script Section 3.4.1)

The first option is to calculate an effect size based on the average data over the largest (positive/negative) cluster. This can be done by averaging the ERP data (per infant and condition) over the channels and time points that comprise the cluster. For the current infant EEG data, Cohen’s d for the average over the positive cluster is 1.057 and Cohen’s d for the average over the negative cluster is − 0.911. This effect size for the average over the cluster best suits the rationale of the cluster-based permutation test. Yet, since clusters do not have an easily defined shape in space and time (see Fig. 3 and Fig. 4), they can be difficult to report comprehensibly which makes it difficult to use this information to inform subsequent studies. Furthermore, whereas we generally expect the observed effect to replicate, we do not necessarily expect to find the exact same cluster shape in space and time. Consequently, an effect size based on the precise cluster might be of limited use for future research.

##### 2.3.3.2 Option 2. Effect size: maximum effect within cluster (Script Section 3.4.2)

A second option is to determine the maximum effect size within the cluster. In other words, we can calculate Cohen’s d for each channel and time point in the cluster and select the largest one. For the current infant EEG dataset, the maximum effect for the positive cluster is 1.064 observed at channel Fp1 at 452 ms and for the negative cluster − 1.063 at channel FC5 at 592 ms. The top panel of Fig. 4 shows the spatial distribution of the effect size for all data points. The advantage of the maximum effect size is that it is precisely determined and easy to report. However, due to random variance in the data, the effect size will fluctuate and the peak effect size will have a positive bias, potentially overestimating the real effect. Consequently, this estimate reflects an upper bound. Moreover, the estimation of where (in space and time) the effect is may be unstable and can be influenced by pre-processing steps such as filtering.

##### 2.3.3.3 Option 3. Effect size: rectangular shape circumscribing the cluster (Script Section 3.4.3)

A third option is to approximate or outline the cluster with a well-defined shape, such as a rectangle, and to calculate the effect size (here Cohen’s d) of the averaged data in this shape. A rectangular shape that fits tightly around a cluster (circumscribed at outside), or a rectangular shape that fits exactly inside the cluster (circumscribed at inside), allows for reporting the corresponding channels and time points precisely. For the current infant EEG data, Cohen’s d for the data averaged over the rectangular shape fitted around the positive cluster (from 338 ms to 594 ms, including channels Fp1, Fp2, F7, F3, Fz, F4, F8, FC5, FC1, FC2, FC6, T7, C3, Cz, C4, T8, FCz, CP5, CP1, CP2, CP6, TP10, P3, Pz, P4, P8, O1, Oz, O2, PO10, TP9) is 0.557 and − 0.730 for the negative cluster (from 544 ms to 704 ms and including channels Fp1, Fp2, F7, F3, Fz, F4, F8, FC5, FC1, FC2, FC6, T7, C3, Cz, C4, T8, FCz, CP5, CP1, CP2, CP6, TP10, P3, Pz, P4, P8, O1, Oz, O2, PO10, TP9). In this example, the rectangular shape happens to encompass many channels and is identical for both clusters, while for other effects cluster results may be more spatially confined. It should be noted that by including data from channels and time points just outside the edges of the original cluster, the effect size estimate might have a slightly smaller value. In contrast to choosing the peak effect in the cluster (Option 2), this estimate provides a lower bound. We elaborate in the section on *Reporting example infant EEG* which effect sizes we consider most useful to report. Note, however, that any of these options, as long as they are transparently communicated, are valid and useful estimates of the observed effect.

### 2.4 Reporting and interpreting developmental EEG data

To support reproducible and replicable developmental EEG research, transparent reporting of details is pivotal. While this is relevant for all aspects of the research, i.e., acquisition, analysis and results, we highlight here what to pay attention to when reporting results of a cluster-based permutation test and how to report effect sizes.

#### 2.4.1 Reporting and interpreting cluster-based permutation tests

For reporting the results of the cluster-based permutation test, one needs to consider that the null hypothesis under which the probability is evaluated states that the data from the different conditions come from the same distribution, and thus is exchangeable. Consequently, a significant result entails that data in different conditions are *not* exchangeable. A valid interpretation of the test for exchangability can therefore be described as “there is a significant difference between condition A and B” (see How NOT to interpret results from a cluster-based permutation test, 2020). In our example of the infant EEG data, we find significant differences between the standard and oddball condition. In contrast, a statement like “We found a significant cluster in area X, between time point A and B” is not correct and should be avoided when reporting results of this nonparametric test, since the cluster is not an explicit part of the tested hypothesis. Cluster-based permutation results themselves do not allow for an inference on precisely where in space, time or frequency an effect occurs (Sassenhagen and Draschkow, 2019). The precise shape of a cluster, and thus its temporal, spatial or spectral extent can differ, depending not only on the data but also on multiple computational parameters (for examples see the section on *Potential factors that influence the formation of clusters*).

However, following the conclusion that the data are different, it is informative to provide a quantitative description of the observed difference. For instance, in our current example the ERP shows a more positive amplitude for oddball stimuli compared to standard stimuli around 400–500 ms after stimulus onset, followed by a more negative amplitude around 550–650 ms at fronto-central channels. Illustrations of the ERPs highlighting the largest cluster(s) can also be very informative, particularly given that clusters typically have a jagged shape (see the top panel in Fig. 3 and Fig. 4 as examples). Providing information on the topographic, temporal, or spectral extent of the cluster, either in the text or by means of illustrations, will help evaluate the outcomes (Sassenhagen and Draschkow, 2019). The current infant EEG results offer a good example of the relevance of interpreting the data rather than the clusters. While two effects were observed (first a positive, followed by a negative condition difference), a more succinct interpretation of the data is not that there were two separate amplitude effects, but rather one latency effect. In other words, the difference in the data suggests that the oddball stimuli elicited a faster negative ERP peak than standard stimuli, suggesting faster processing of the oddball compared to the standard stimuli in 9-month-old infants. Having established that there is a significant difference in the conditions, one might in this case consider to follow up on the interpretation of the difference by explicitly testing for peak latency differences.

#### 2.4.2 Reporting and interpreting effect sizes

As we discussed above, for EEG data comprising many channels and time points, multiple quantifications of the effect size are possible. We argue that reporting both the maximum effect within a cluster (Option 2) and the effect size of the average over the rectangular shape surrounding a cluster (Option 3) contribute most efficiently to advance future research. There are two reasons for this. First, together these describe an upper and lower bound of the effect size estimate. Second, both can be reported precisely and in a comprehensible manner, by listing exactly which channels and time points they are based on. The range of the estimated effect size, the peak, and the spatial and temporal extent provide optimal prerequisites to guide the study and analysis designs for follow-up research.

In addition to reporting detailed outcomes of a developmental EEG study in a publication, providing material, such as the EEG data in BIDS format, the code used for analysis, and also the stimulus material, further fosters informed follow-up research (see Fig. 1). This contributes to hypotheses formation and properly powered designs for new data collection. It also allows for learning from existing analysis code and reducing the number of analysis choices to be considered for future analysis, and for re-analyzing existing EEG datasets to address new research questions.

## 3 Conclusion

Being able to evaluate the quality of developmental EEG research and estimate how robust and large an observed effect in developmental EEG data is, builds the foundation for further advances in developmental cognitive neuroscience research. Given how costly data collection with developmental populations is, basing subsequent research on false discoveries means unnecessary waste of substantial resources. In this paper, we highlighted how using the cluster-based permutation test, and estimating and reporting effects sizes can contribute to more reproducible research. We also described how infant EEG data can be transformed into BIDS format, following FAIR data sharing principles. To make these approaches more accessible to the developmental cognitive neuroscience community, we provide example analysis code and an infant EEG dataset which are made publicly available. We hope to thereby contribute to the joint effort of the developmental cognitive neuroscience community (e.g., Klapwijk et al., 2020) in maximizing the quality and information gain of developmental EEG research.

## Declaration of Competing Interest

The authors declare that they have no known competing financial interests or personal relationships that could have appeared to influence the work reported in this paper.

## Data Availability

This study is based on an existing, anonymized developmental EEG dataset from 59 infants. The data and stimulus material are organized according to the BIDS standard and shared in the Donders Repository (https://doi.org/10.34973/gvr3–6g88). We also provide the MATLAB analysis scripts using the open source FieldTrip toolbox. The code was co-developed and is maintained by the authors on GitHub (https://github.com/Donders-Institute/infant-cluster-effectsize). Intermediate and final results of the analysis are also shared in the Donders Repository (https://doi.org/10.34973/g4we-5v66), including a copy of the code as used for this manuscript.

## Footnotes

1 We adopt the definition of replication and reproducibility as used in Peng (2011) who regards reproducibility as spectrum leading up to the gold standard of full replication.
